# 

**Published:** 1883-07

**Authors:** 


A MONTHLY JOURNAL.

DEVOTED TO

MEDICINE, SURGERY, OBSTETRICS, DENTISTRY, PATHOLOGY AND POPULAR SCIENCE.

---

**W. C. BARRETT, M. D., D. D. S.,**

EDITOR.

COLUMBIA PUBLISHING COMPANY.

**$2.50 Per Annum in Advance. - - Single Copies, 25 Cents.**

Entered at the Post-Office, Buffalo, N. Y., as Second-Class matter.

